# Discrimination of standing postures between young and elderly people based on center of pressure

**DOI:** 10.1038/s41598-020-80717-z

**Published:** 2021-01-08

**Authors:** Kimiya Fujio, Yahiko Takeuchi

**Affiliations:** 1 Department of Rehabilitation for Movement Functions, Research Institute of National Rehabilitation Center for Persons with Disabilities, Saitama, Japan; 2 Department of Rehabilitation Medicine, Chiba University Hospital, Chiba, Japan

**Keywords:** Motor control, Neural ageing

## Abstract

Posturography is utilized to assess the influence of aging on postural control. Although this measurement is advantageous for finding group-level differences between the young and the elderly, it is unclear whether it has the potential to differentiate elderly individuals who are affected by various impacts of aging. The purpose of this study was to determine the utility of posturography to discriminate elderly individuals from young adults. We investigated the performances of the random forest classifiers constructed from center of pressure (COP) indices for discriminating standing postures between healthy elderly and young people. Postural sways in 19 young and 31 community-dwelling elderly participants were measured using force plates in 4 standing conditions: bipedal standing, standing on a narrow base, standing on foam rubber, and standing with eyes closed. We further verified the informative predictors that contributed to the prediction model. As the results, the classifier based on the COP indices for standing on foam rubber showed the best performance (accuracy: 93.4%, sensitivity: 94.4%, specificity: 93.6%, area under the curve of receiving operator characteristics: 0.95), followed by the classifier for standing with eyes closed. The informative predictors varied depending on the postural conditions. Our findings demonstrated the potential of posturography for identifying elderly postures. The evaluation of sensory re-weighting using the appropriate COP indices would be a useful clinical tool for detecting the progress of aging on postural control.

## Introduction

Balance impairment is a critical cause of falling in elderly people. Falling is one of the major geriatric syndromes that leads to bone fractures, head trauma, and other health problems. It has been demonstrated that the influence of aging on standing posture is characterized by increased co-contraction in the lower-limb muscles^1^, greater stiffness of the ankle joint^2^, greater physiological tremor leading to force fluctuations^3^, less flexible coordination of joint movement^4^, and changes of sensory preference^5^. These effects of aging can be attributed to a reorganization of the neurological system, which includes higher corticospinal excitability^6^ and a lack of reflex modulation^7^, as well as to muscle weakness. The alterations of the neural bases for postural control make it difficult for the elderly to tune their postures for context-dependent requirements.

Age-related changes in standing balance are typically assessed with several clinical tests, such as the Sensory Organization test, the Functional Reach test, the Timed Up and Go test, and the motor perturbation test^8–11^. Posturography of quiet standing is one of the common tools for quantifying postural sway in clinical practice. The center of pressure (COP) is generally evaluated in temporal, topographic, and frequency domains, and not all COP indices in each domain are affected by aging. Several studies have investigated which COP indices can be used detect the impact of aging on standing balance^12–14^. A set of multiple indices was thought to be more useful than a single index for assessing postural steadiness^15,16^. The selection of more sensitive indices from multivariate candidates could make this assessment more accurate.

The question of which postural condition is evaluated is also important. Measurements of the COP with sensory and/or mechanical constraints highlight the age-related changes in posture. For example, when visual information was occluded, postural sway in the elderly became faster and larger in the mediolateral (ML) direction, while young people were not affected^17^. This result was potentially explained by the finding that elderly people have insufficient sensory re-weighting which is used to adjust their postures according to the sensory context^18,19^. Elderly postures are also highly susceptible to restriction of the base-of-support area on which more elaborate joint configurations are required^20,21^. A moderate challenging posture would amplify the impacts of aging even before postural steadiness decreases.

Combining those approaches, numerous studies revealed group-level differences in the COP indices between elderly and young people^13,22–25^. However, there is little evidence regarding the sensitivity of the COP for the extent of aging in elderly individuals. Given that the impacts of aging on posture vary widely among elderly people^26^, it may be reasonable that some elderly individuals do not show clear differences from young individuals^27,28^. Although a more sensitive assessment for such individuals would be valuable in terms of the prevention of balance impairment, it is unclear whether the COP could be used to differentiate elderly individuals who do not have obvious postural changes due to aging. A classifier, based on a machine learning method, using informative features selected from the multiple COP indices could be useful for this differentiation. When a particular individual is tested, such a classifier would indicate whether or not the individual’s standing posture is categorized as being in an elderly group, which provides definitive information; a comparison with normative benchmarks in each COP index would also be informative. The purpose of this study was to clarify whether COP could be used to discriminate elderly postures from young postures regardless of the extent of aging, and, if the COP can do so, we attempted to identify which indices are the most informative. In this process, the utility of COP as an indicator of the progress of aging on postural control would be supported.

In contrast to previous studies that compared group-level differences, we investigated how accurately elderly individuals were identified from among unlabeled participants by using multiple COP indices in different postural conditions. For this purpose, we used a random forest (RF), which is a tree-based ensemble approach based on machine-learning algorithms. First, each participant’s COP was measured in four different postures with sensory and mechanical constraints to amplify the differences between young and elderly postures. From the time-series of the COP excursions, 69 COP indices were calculated in each condition, and subsequently, the RF classifiers were constructed using those indices in the four conditions. We further searched for informative predictors that can be used as sensitive COP indices for age-related changes based on the permutation importance measure. Finally, we compared the performances of these classifiers by using the confusion matrix in each condition.

## Methods

### Participants

Nineteen healthy young adults and 33 healthy elderly adults participated in this study. The data of three participants (one young person, two elderly people) were excluded because of a recording error for one young person and neurological signs in two elderly people. Thus, the remaining data of 49 participants (Young: 13 females, age 20.3 ± 0.6 years, Elderly: 15 females, age 73.3 ± 3.9 years) were analyzed. All 49 participants had no known major orthopedic or neuromuscular diseases and no physical disabilities and were able to stand unaided. They provided written informed consent in accordance with the Declaration of Helsinki. This study was approved by the ethics board of Chiba Prefectural University of Health and Sciences.

### Measurements and tasks

Ground reaction forces were collected from a force plate system which digitized the data sequentially to obtain COP coordinates in anteroposterior (AP) and ML directions with a sampling frequency of 100 Hz (BR400600, AMTI, Inc). The force data were filtered by a second-order, zero-phase-lag Butterworth low-pass filter with a cut-off frequency of 10 Hz. The participants were instructed to keep standing on one- or two-force plates according to the experimental conditions. The participants gazed at a visual target that was displayed 1.5 m in front of their standing position at eye level. They were instructed not to move intentionally during the measurement. We also recorded 29 reflective-marker positions which were attached to the participant’s whole body as a Helen Hayes-marker set using eight infrared cameras (Mac3D system, Motion Analysis, Corp). In this study, however, only the force plate data were analyzed.

Measurements were performed in 10 trials for each participant. Participants were instructed to stand in 5 different postures with eyes opened and eyes closed, respectively: bipedal standing, standing on a narrow base, standing on foam rubber, standing in tandem position, and single-leg standing. We analyzed 4 of the 10 trials as the experimental conditions in this study: standing with eyes opened (Normal), narrow-base standing with eyes opened (Narrow), standing on a foam rubber with eyes opened (Foam), and standing with eyes closed (EC). The data for tandem and single-leg standing were excluded because not all elderly people could keep standing for the full recording time in both conditions. In the Normal, Foam, and EC conditions, two force plates were used for measurements. Participants stood with their right and left heels separated by 15.0 cm and aligning with parallel lines in the AP direction on each plate. In the Narrow condition, participants stood with both feet closed on one force plate. In the Foam condition, participants stood on foam rubber which was composed of natural rubber with a tensile strength of 2.1 kgf/cm^2^, a density of 0.06 g/cm^3^, an elongation stretch percentage of 110%, and a thickness of 3.5 cm.

Every trial was recorded for 60 s, and the first 10 s were excluded in the follow-up analysis. All participants were able to keep standing independently during recording in all conditions. Measurements were performed after practice so that participants understood the test position. The order of the 10 trials (5 postures, 2 eye-conditions) was randomized for each participant.

### Data processing and statistical analysis

With reference to previous studies^12–14,29–31^, 69 indices in planar and AP/ML directions were computed from the COP time-series (Supplementary Table 1). In this study, the mean value of COP in the AP and ML planes was subtracted from the entire time series because it was inevitable that the positions of the participants’ feet would misalign when the rubber mat was used in the Foam condition.

RF, which is a tree-based ensemble approach, was used as a classifier for young and elderly postures. Two parameters, i.e. the number of trees and the number of indices, had to be tuned to construct each model^32^. The appropriate number of trees was assessed from 1 to 5000, 10,000 15,000 and 20,000 and the lowers point at which the variance of the out-of-bag (OOB) errors reached a plateau was determined in each condition (Supplementary Fig. 1). We set 5,000 trees as the fixed value with no pruning in this study. For the number of indices, the best ratio from 10 to 100% in steps of 10% of the 69 COP indices that minimized the value of the OOB error was determined for each training dataset. The participants were split between 48 training datasets and 1 test data for the leave-one-out method, and in total 49 datasets were built. The RF classifier was constructed by bootstrap aggregation from 48 training datasets, and the resultant data were used to compute the OOB error. After the number of indices was determined, the RF classifier was constructed from the training data in every dataset and its accuracy was examined using test data. Finally, the confusion matrix was built to assess the performance of the RF classifier. The prediction accuracy, sensitivity, specificity, and area under the curve (AUC) of the receiving operator characteristics were calculated in each condition as the performance parameters. We further searched for informative predictors for the RF classifier with permutation importance, which is one of the variable importance measures based on the prediction accuracy of the permuted predictor^33^. All analyses were performed using MATLAB software.

We further performed a hierarchical cluster analysis of 69 COP indices in order to categorize the informative predictors into subgroups. This analysis was expected to exhibit the similarities among 69 COP indices and to determine which domains of the COP are sensitive to age-related changes. The distance between two indices was defined by 1—Spearman’s correlation coefficient. The average method was applied to all possible pairs for the distance between two clusters.

For a comparison of the performance of the classifiers, the accuracy, sensitivity, specificity, and AUC of each were calculated in the four conditions. All COP indices were compared between the young and elderly groups using the Wilcoxon rank sum test after the Shapiro–Wilk normality test. Statistical significance was set at α = 0.05, and the results are presented as means ± standard errors (Supplementary Table 2).

## Results

### Comparison of the COP trajectories and time-series

**Figure 1:**
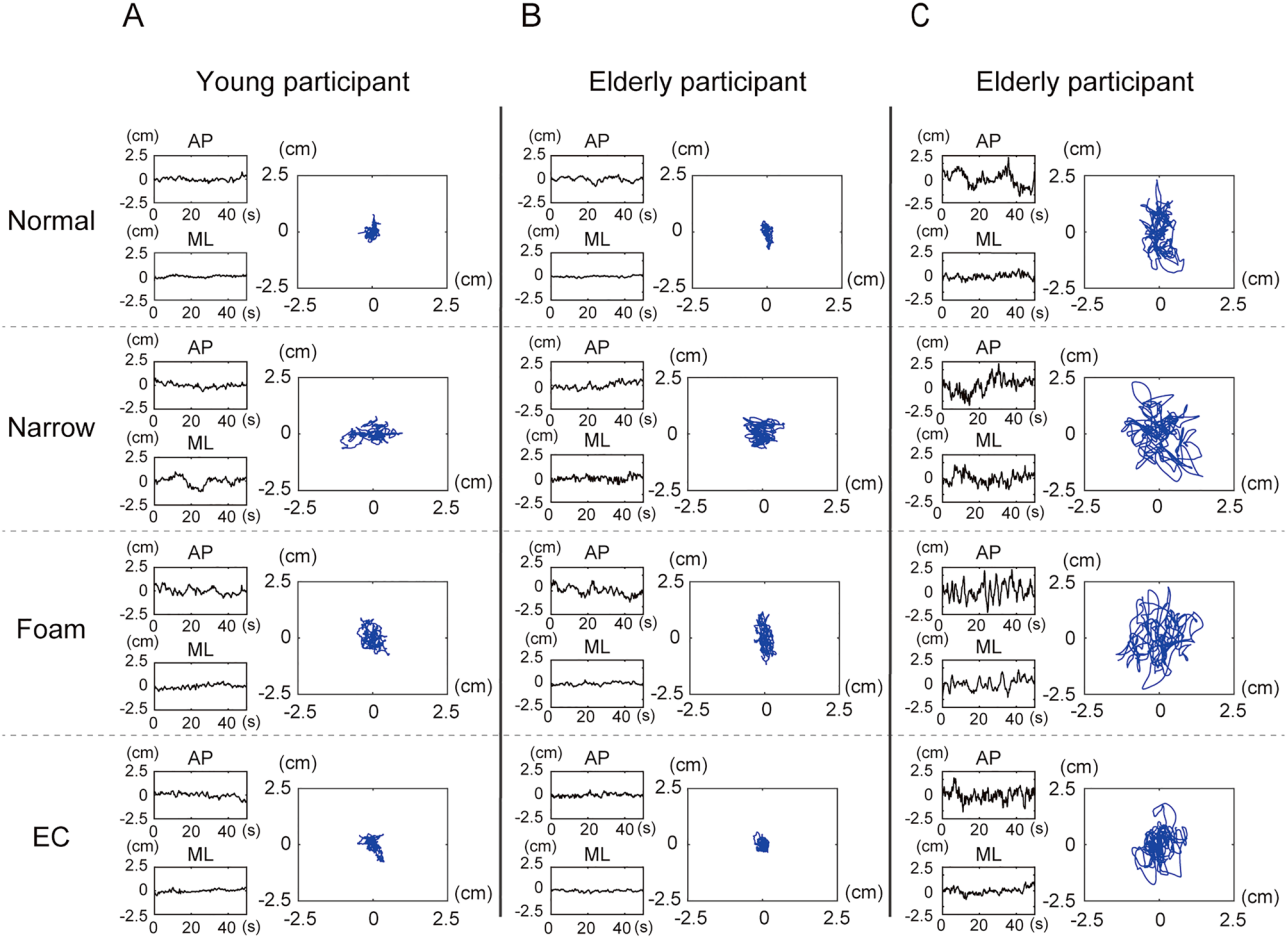
Representative data of time series of the COP displacements in the anteroposterior and mediolateral directions, and the COP trajectories in the planar plane for one young (**A**) and two elderly (**B**,**C**) participants. Normal: bipedal standing, narrow: standing on narrow base of support, foam: standing on foam rubber, EC: standing with eyes closed.

The COP trajectories and time-series of three representative participants (one young person, two elderly people) in the different conditions are shown in Fig. 1. The difference between the young participants and one elderly participants (A vs. C) could be observed in terms of a larger 2D excursion in all conditions. For the other elderly person, however, COP excursion was similar to the young participants according to visual inspection (A vs. B). As we postulated, there was an elderly person who exhibited postural sway characteristics similar to those of the young individuals.

### Performances of the RF classifiers

As a result of the parameter tuning, the number of trees was set to 5000. Table 1 illustrates the performances of the RF classifiers in four conditions. The discrimination accuracy was different among the four conditions despite applying the same procedure for the RF. The best accuracy was demonstrated in the Foam condition (93.4%), followed by the EC condition (89.8%). The sensitivity, specificity, and AUC were concomitantly high values in these two models. On the other hand, in the Normal and Narrow conditions, the classification accuracy was lower than in the other two conditions (75.6% and 67.4%, respectively). The performance of the RF classifier in the Narrow condition was the worst of all the conditions.

**Table 1:** Performance of RF classifiers in 4 different conditions.

	Normal	Narrow	Foam	EC
Accuracy	75.6%	67.4%	93.4%	89.8%
Sensitivity	55.6%	33.4%	94.4%	83.3%
Specificity	87.1%	87.1%	93.6%	93.6%
AUC	0.85	0.70	0.95	0.90

*AUC* area under the curve of receiving operator characteristics.

### Informative predictors for identification of elderly postures

**Figure 2:**
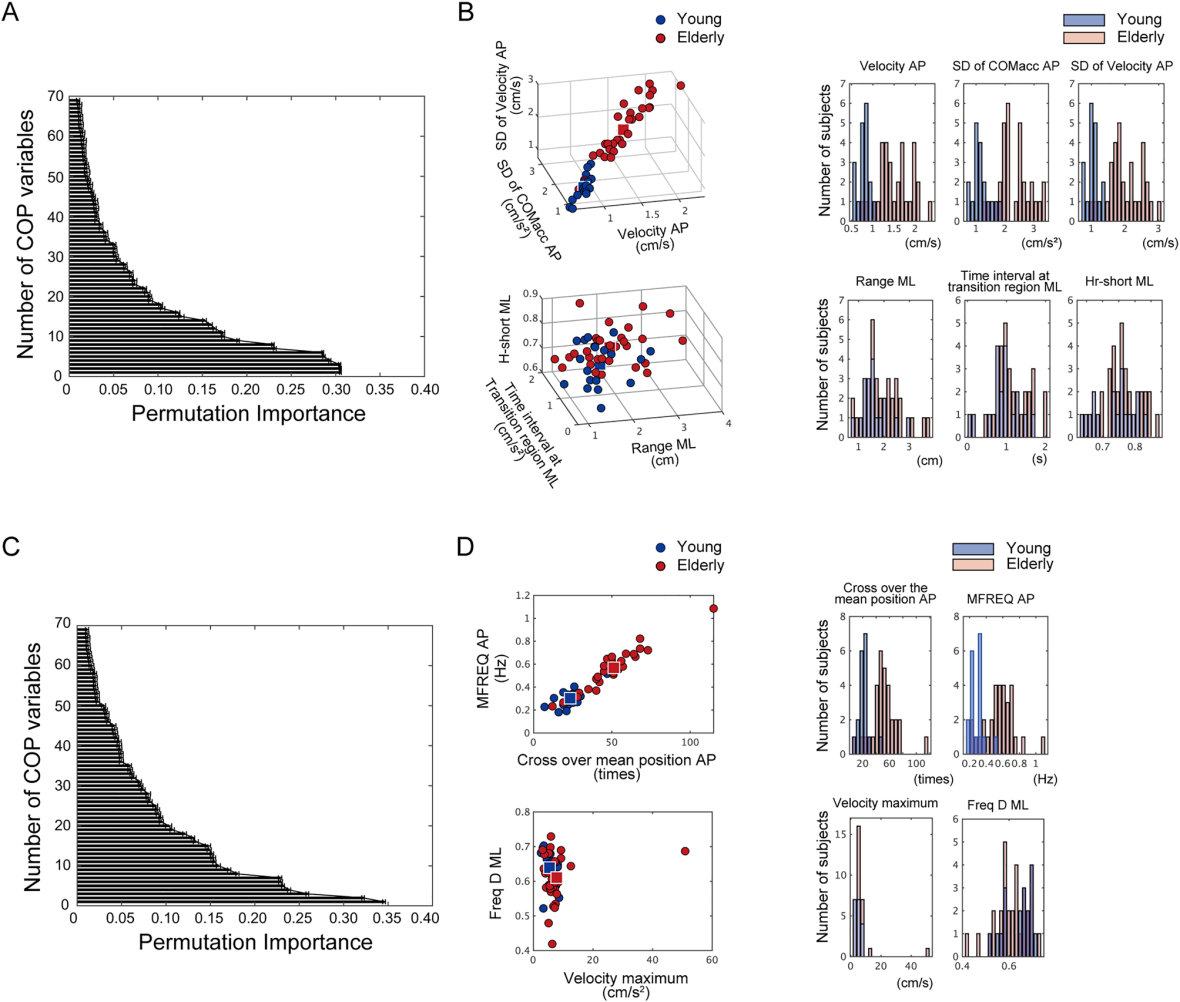
The informative predictors extracted by the random forest in the Foam (upper row) and EC conditions (lower row) for discriminating young and elderly postures. (**A**) Variable importance for the COP indices in the Foam condition. (Standard errors are shown). (**B**) Scatter plots and histograms of the informative and un-informative predictors in the Foam condition (upper row; informative predictors, lower row; un-informative predictors, left; scatter plots, right; histograms). Three informative predictors were extracted in the Foam condition. The same number of uninformative predictors as informative predictors were chosen in the order beginning with the lowest value of the permutation importance. Square symbols in scatter plots represent the average values. (**C**) Variable importance for the COP indices in the EC condition. (**D**) Scatter plots and histograms of the informative and uninformative indices in the EC condition (upper row; informative indices, lower row; un-informative indices). Two informative and uninformative predictors were plotted in the Foam condition.

Regarding the high performance of classifiers in the Foam and the EC conditions, the informative predictors were determined based on the variable importance measure (Fig. 2A,C). To visualize the distribution of informative predictors in low-dimensional space, we set the cut-off value at 0.3. In the Foam condition, three indices, namely the COP velocity in AP, the standard deviation (SD) of the center of mass (COM) acceleration in AP, and the SD of the COP velocity in AP were extracted. In the EC condition, the number of crossings of the mean-zero position in the AP (Mean-cross AP) and the mean frequency of rotational COP movement on the circle with a radius of the root mean square distance (MFREQ AP) were selected. Figure 2B,D show scatter plots and histograms of the informative and uninformative predictors which extracted the same number of indices from the lowest value of variable importance. The distributions of the informative predictors could be split among the different age groups even in low-dimensional planes in contrast to those of unimportant indices. The same trends could be observed from the histograms: the bimodal distribution of the informative predictors and the monomodal distribution of the uninformative predictors.

**Figure 3:**
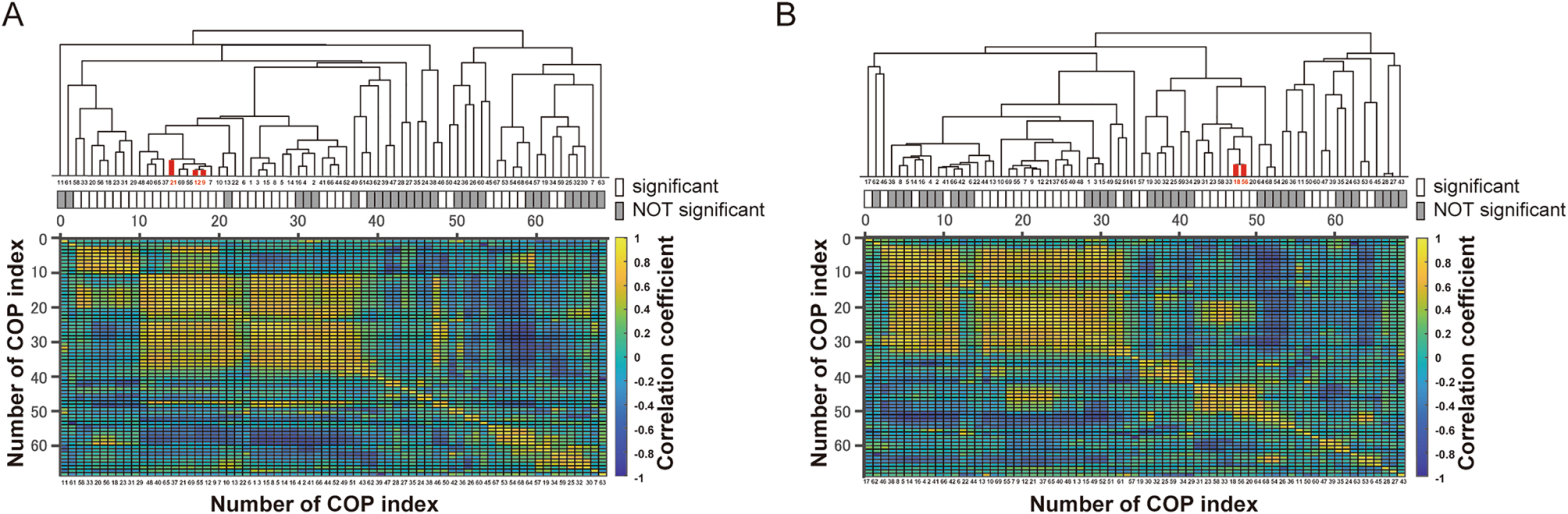
Correlation matrix and the dendrogram of all pairwise COP indices. The correlation matrix and the dendrogram by the hierarchical cluster analysis for 69 COP indices in the Foam (**A**) and EC (**B**) conditions. Thick red lines represent the informative predictors in each condition (the Foam condition; Velocity AP, SD of COMacc AP, and SD of Velocity AP extracted in the Foam, the EC condition; Mean-cross AP, MFREQ AP). The correlation coefficients between 2 indices for all combinations are pictured with the color map, which was used as the distance.

The similarities among indices were confirmed by cluster analysis using the correlation coefficient of 69 indices (Fig. 3). The correlation matrix and the dendrogram demonstrated that three informative predictors in the Foam conditions were allocated in the same cluster, which was relevant to the velocity. In the EC condition, Mean-cross AP and MFREQ AP, which were distinct from the velocity-related cluster, were also categorized in the same cluster.

We further compared the average values of all indices in each condition to test the group-level differences. Similar to the previous studies, significant group differences between the young and elderly participants were found in a number of indices (22 in the Normal condition, 31 in the Narrow condition, 41 in the Foam condition, and 35 in the EC condition; Supplementary Table 2). All informative predictors in both the Foam and EC conditions were significantly different, which supported the assumption that they were sensitive to aging.

## Discussion

The present study demonstrated that the standing posture of elderly individuals could be discriminated by the COP indices, especially when they were measured using foam rubber, from the standing posture of young people with high accuracy. The velocity-related indices, including the COP velocity, the SD of the COM acceleration, and the SD of the COP velocity in AP direction, were extracted as informative predictors for detecting aging in the Foam condition. The COP-based classifier from the standing with eyes closed condition also performed this discrimination well, along with the different informative predictors from the standing on foam rubber condition. The statistical analysis revealed significant differences in a number of indices between the young and elderly groups in all conditions, regardless of the performance of the RF classifier, a finding that supported the results of previous studies^13,14^. These findings suggest that the COP has the potential to be used to evaluate the impact of aging on postural control at both the individual and group levels, and the manipulation of proprioceptive and visual inputs during measurement could highlight it.

### The utility of assessments of sensory re-weighting for detecting age-related changes in standing posture

The central nervous system organizes the visual, vestibular, and somatosensory inputs for stable standing. It has been proposed that an assessment of the re-weighting be conducted for patients with neurological disease or for the forecasting of future falling^34–38^. For example, Fujimoto et al. compared the diagnostic accuracy of the COP parameters for peripheral vestibular patients between normal standing and standing on foam rubber^36^, and that population of patients was identified with high accuracy by using Romberg’s ratio of the COP velocity measured on a foam rubber mat, regardless of the patients’ age. They suggested that sensory manipulation in the COP measurement was advantageous for the preliminary assessment of vestibulopathy.

With regard to the risk of falling, the use of posturographic data recorded on a compliant surface was able to differentiate the individuals who had had a fall experience^37^. In that study, the principal components of the 17 COP indices were distinguishable among non-fallers, rare fallers, and frequent fallers. In support of that previous research, our present results demonstrated that the evaluation of sensory re-weighting is valuable for discriminating the healthy elderly from young people. The results imply that the elderly cannot compensate for the distortion of the proprioceptive or visual input using other sensory systems. This finding may remain controversial because healthy elderly people may be capable of sensory re-weighting at the same level as the young when only one sensory input is removed^19,39^. We postulate that a selection of the COP indices was also important for detecting age-related changes that do not cause a fall directly. In the present analysis, since the informative predictors were automatically selected from many candidates, the elderly individuals could be separated from the young with high accuracy.

In general, the postural sway in the elderly is larger, faster, and more random than that in young people. We observed that the velocity-related indices were the most informative predictors in the RF model in the Foam condition. This finding is in line with the abundant literature showing that the COP velocity is significantly increased in the elderly compared to the young^13,14,23,38^. Since the velocity-related indices are averaged on time-series data, they are far more stationary than the spatial indices. In addition, the COP velocity reflects the body’s acceleration, which is the controlled variable for static standing^40^, when approximated with the inverted pendulum model. These indices can therefore be appropriate predictors of the elderly posture.

Notably, the informative predictors were not consistent between the Foam and EC conditions. The Mean-cross AP and MFREQ AP, which are calculated based on the mean COP position, meant that the COP excursion of the elderly tended to move away from the mean position with high-frequency postural sway when their vision was occluded. The inconsistency of informative predictors was also confirmed by the dendrogram in which those predictors were assigned in different clusters. The difference in informative predictors may reflect distinctive responses of sensory re-weighting against proprioceptive and visual disturbance.

### Discrepancy between the COP indices for age-related changes and for fall prediction

On the other hand, the above-described informative indices were not consistent with the markers of future falls. It is well established that changes in velocity and frequency in the ML direction are relevant to the risk of falling^17,20,33,41,42^, while all of the informative predictors in the present study were categorized in the AP direction. As supported by several studies^1,43,44^, it is possible that postural sway in elderly people can be altered in the AP direction. We concluded that alteration in the AP direction emerges even in elderly persons who do not indicate an obvious postural change due to aging.

The preservation of the ML stability in the elderly participants was also confirmed by the results in the Narrow condition, in which the postural sway in the elderly was similar to that in the young people, regardless of any restriction of stance width in the ML. For the purpose of discriminating between the postures of the young and the healthy elderly, the COP indices in the AP direction were more appropriate rather than the ML, even though significant group differences were observed in both directions.

### Possible mechanisms

Such differences in the COP indices due to aging can be attributed to changes in the sensory receptors and central processing. Standing posture in the AP direction is mainly controlled by both the ankle and hip joints. The joint coordination between the hip and ankle joints varies due to aging, which induces a larger COM acceleration in elderly people^45^. It is thought to be related to alterations of the sensitivity of the muscle spindle^46,47^, the excitability of the spinal reflex, and structural changes in the cortical and subcortical regions^48,49^. Those changes cause a decline of sensory re-weighting, which is linked to increased co-contraction and decreased joint coordination. The manipulation of the proprioceptive and visual input would highlight those deficits and result in the amplification of the differences between young and elderly postures. Future studies are expected to investigate the causal relation between the results of the discrimination using the COP indices and physiological changes.

### Study limitations

There is a limitation regarding the implications of the variable importance measure. We set the cut-off value for the permutation importance to visualize their distribution in the low-dimensional space. This could not conclude whether or not other variables are important. It is possible that the correlated predictors bias the value of variable importance measure^33^, although we illustrated that the young and elderly postures were partitionable based on this measure. In future studies, novel methods should be applied to determine the most important COP indices for assessing the progress of aging on standing posture.

## Conclusions

Our study demonstrated the utility of posturography for identifying the COPs of the healthy elderly. The RF classifier showed high accuracy in 2 conditions, namely standing on foam rubber and standing with eyes closed. The COP indices that were informative for that discrimination depended on the postural condition: the velocity-related indices in the Foam condition and the mean frequency and amplitude of postural sway relative to the mean-COP position in the EC condition. These results suggest that the age-related changes of standing posture can be captured with the appropriate COP indices measured under sensory-manipulated conditions. Combined with the conventional analysis comparing with a normative benchmark, the classification method described herein can be used to detect postural changes in elderly individuals. Further studies are necessary to establish objective indices that can identify individuals who are at high risk of falling, toward the indices’ use as a clinical tool.

## Supplementary Information

Supplementary Information
