# Supplementary material for: Discrimination of standing postures between young and elderly people based on center of pressure
Source: Sci Rep. 2021 Jan 8;11:195. doi: 10.1038/s41598-020-80717-z (PMC7794377; doi:10.1038/s41598-020-80717-z)
Supplement: Supplementary file 1 — Supplementary Information [file 41598_2020_80717_MOESM1_ESM.pdf]

Supplementary Information

Article in *Scientific Reports*

Discrimination of Standing Postures between Young and Elderly People Based on Center of Pressure

Kimiya Fujio*, PhD; Yahiko Takeuchi, PhD

*Correspondence: Kimiya Fujio, PhD, Department of Rehabilitation for Movement Functions, Research Institute of the National Rehabilitation Center for Persons with Disabilities, Saitama, Japan

Address: 4-1 Namiki-cho, Tokorozawa city, Saitama, 359-8555, Japan

Phone: +81-(0)04-2995-3100

E-mail: fujiokimiya@yahoo.co.jp

Supplementary Table 1. Glossary of the 69 COP indices

No.	Name	Description
1	Range AP	Distance between maximum deviation in AP
2	Range ML	Distance between maximum deviation in ML
3	SD of AP sway	Standard deviation of AP sway
4	SD of ML sway	Standard deviation of ML sway
5	Area	95% confidence of ellipse
6	Velocity maximum	Maximum velocity in planar plane
7	Total distance	Total distance of COP trajectory
8	Area circle	95% confidence of circumference
9	Velocity AP	Mean velocity in AP
10	Velocity ML	Mean velocity in ML
11	Angle	Angular deviation from the AP line
12	SD of Velocity AP	Standard deviation of the velocity in AP
13	SD of Velocity ML	Standard deviation of the velocity in ML
14	Sway area	Triangle area enclosed by mean COP position and 2 consecutive points per unit of time
15	Major	Length of the longitudinal axis of 95% confidence ellipse
16	Minor	Length of the short axis of 95% confidence ellipse
17	Eccentricity	Eccentricity of 95% confidence ellipse
18	MFREQ AP	Mean Frequency with a circular motion with a radius of mean AP
19	MFREQ ML	Mean Frequency with a circular motion with a radius of mean ML
20	F-dim	Fractal dimension
21	SD of COMacc AP	Standard deviation of acceleration of COM in AP
22	SD of COMacc ML	Standard deviation of acceleration of COM in ML
23	Slope-LF AP	Slope of regression line at low frequency band of power spectral density in AP
24	Slope-HF AP	Slope of regression line at high frequency band of power spectral density in AP
25	Slope-LF ML	Slope of regression line at low frequency band of power spectral density in ML
26	Slope-HF ML	Slope of regression line at high frequency band of power spectral density in ML
27	Intersect-PSD AP	Intersection of regression lines at low and high frequency band of PSD of AP in log-log plot

No.	Name	Description
28	Intersect-PSD ML	Intersection of regression lines at low and high frequency band of PSD of ML in log-log plot
29	PF50 AP	50% power frequency in AP
30	PF50 ML	50% power frequency in ML
31	PF95 AP	95% power frequency in AP
32	PF95 ML	95% power frequency in AP
33	CF AP	Centroidal Frequency in AP
34	CF ML	Centroidal Frequency in ML
35	FD AP	Frequency Dispersion in AP
36	FD ML	Frequency Dispersion in ML
37	D-short AP	Diffusion coefficient at short-term region in AP
38	D-long AP	Diffusion coefficient at long-term region in AP
39	Critical-Δtime AP	Time interval at the critical point in AP for the stabilogram-diffusion plot
40	Critical-mm AP	Mean square COP displacement in AP at the critical point for the stabilogram-diffusion plot
41	D-short ML	Diffusion coefficient at short-term region in ML
42	D-long ML	Diffusion coefficient at long-term region in ML
43	Critical-Δtime ML	Time interval at the critical point in ML for the stabilogram-diffusion plot
44	Critical-mm ML	Mean square COP displacement in ML at the critical point for the stabilogram-diffusion plot
45	Hr-short AP	Scaling exponent of mean square COP displacement and time interval on log-log plot for short-term region in AP
46	Hr-long AP	Scaling exponent of mean square COP displacement and time interval on log-log plot for long-term region in AP
47	Intersect-Δtime AP	Time interval at the at the transition region in AP for the log-log plot of the stabilogram-diffusion plot
48	Intersect-mm AP	Mean square COP displacement at the transition region in AP for the log-log plots of the stabilogram-diffusion plot
49	Hr-short ML	Scaling exponent of mean squared COP displacement and time interval on log-log plot for short-term region in ML
50	Hr-long ML	Scaling exponent of mean squared COP displacement and time interval on log-log plot for long-term region in ML

No.	Name	Description
51	Intersect-Δtime ML	Time interval at the at the transition point between short- and long-term region on log-log plot in ML
52	Intersect-mm ML	Mean square COP displacement at the transition point between short- and long-term region on log-log plot in ML
53	MT3	Mean time interval between successive peaks on sway-density curve at radius = 3
54	MP3	Mean peak value on sway-density curve at radius = 3
55	MD3	Mean distance between successive peaks on sway-density curve at radius = 3
56	Mean-cross AP	The number of crosses of mean COP position in AP
57	Mean-cross ML	The number of crosses of mean COP position in ML
58	Zero-cross AP	The number of zero crosses of COP velocity in AP
59	Zero-cross ML	The number of zero crosses of COP velocity in ML
60	Alpha AP	Shape parameter of Gamma distribution approximated the distribution of time interval for crossing zero of COP velocity in AP
61	Beta AP	Scale parameter of Gamma distribution approximated the distribution of time interval for crossing zero of COP velocity in AP
62	Alpha ML	Shape parameter of Gamma distribution approximated the distribution of time interval for crossing zero of COP velocity in ML
63	Beta ML	Scale parameter of Gamma distribution approximated the distribution of time interval for crossing zero of COP velocity in ML
64	LNG-area	Total distance of COP trajectory divided by 95% confidence ellipse area
65	Total power AP	Total power in AP
66	Total power ML	Total power in ML
67	Mean-MT	Average of mean time interval on sway-density curve from radius = 2 to 5
68	Slope-MP	Slope of regression line on MP-Radius plot between radius = 2 to 5
69	Mean-MD	Average of mean distance on sway-density curve from radius = 2 to 5

AP, Anteroposterior direction; ML, Mediolateral direction; COP, Center of Pressure; COM, Center of Mass

Supplementary Figure 1. Changes of out-of-bag errors based on the number of trees

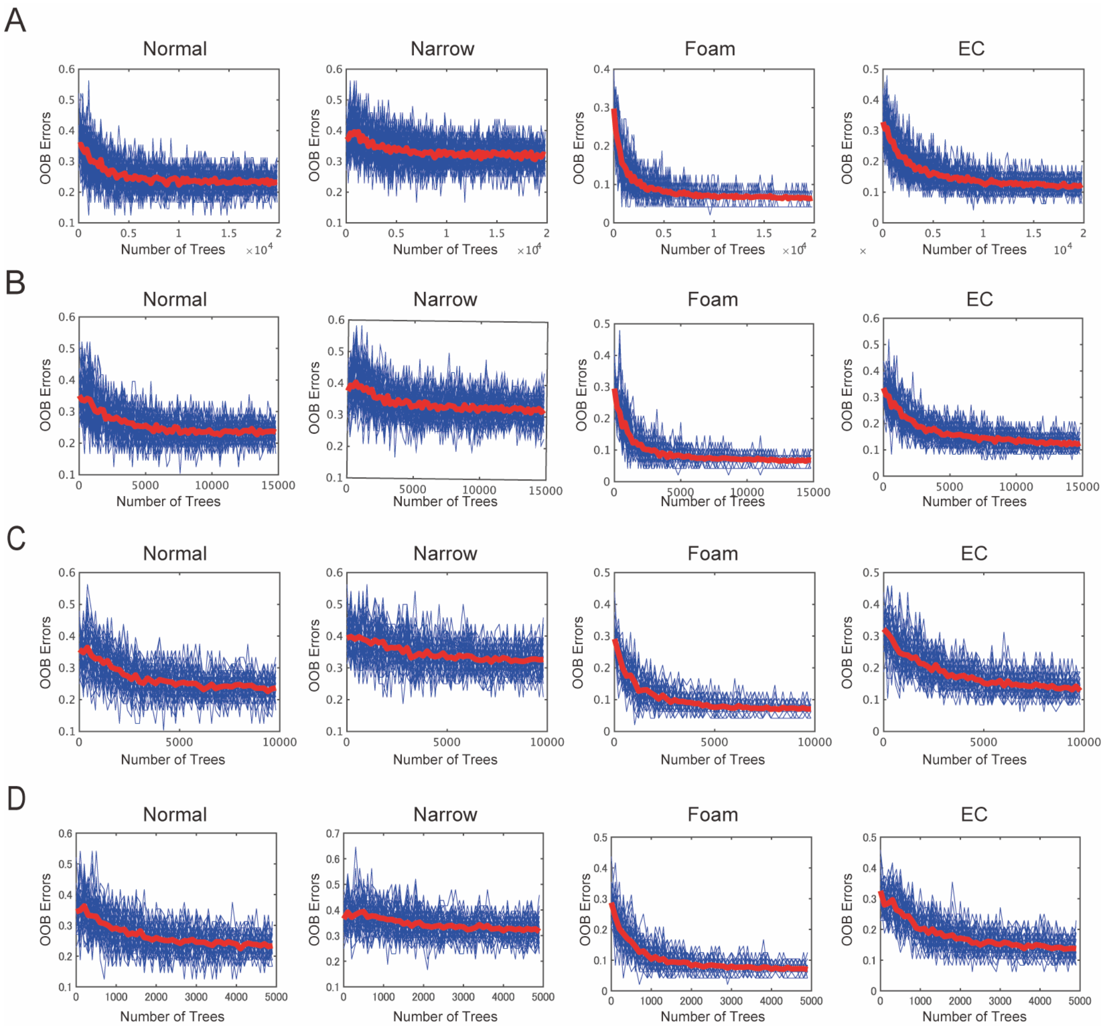

To determine the number of trees using in Random Forest, transition of out-of-bag (OOB) errors were displayed based on the maximum number of trees. Four different maximum numbers were test: A. 20,000; B. 15,000; C. 10,000; D. 5,000. Thick red lines represent the average value of the OOB errors by 49 classifiers in each number of trees. All COP indices were used to construct each classifier for this validation. The average lines reache plateau approximately 5,000 of trees in all condition, except for the case of 5,000 trees for the maximum number (D).

Supplementary Table 2. Statistical differences of the COP indices between young and elderly people

No.	Name	Normal				
		Mean (Young)	SE (Young)	Mean (Elderly)	SE (Elderly)	p-value
1	Range AP (cm)	1.98	0.14	2.21	0.15	0.378
2	Range ML (cm)	1.10	0.11	1.12	0.07	0.844
3	SD of AP sway (cm)	0.38	0.03	0.43	0.03	0.671
4	SD of ML sway (cm)	0.19	0.02	0.21	0.02	0.276
5	Area (cm^2)	1.06	0.14	1.46	0.21	0.462
6	Velocity maximum (cm/s)	5.97	0.61	4.71	0.28	0.087
7	Total distance (cm)	36.97	1.46	49.68	2.53	< 0.001
8	Area circle (cm^2)	1.72	0.19	2.33	0.39	0.732
9	Velocity AP (cm/s)	0.54	0.02	0.82	0.05	< 0.001
10	Velocity ML (cm/s)	0.39	0.02	0.40	0.02	0.926
11	Angle (degree)	11.87	2.62	9.58	1.96	0.437
12	SD of Velocity AP (cm/s)	0.70	0.03	1.06	0.06	< 0.001
13	SD of Velocity ML (cm/s)	0.55	0.03	0.52	0.02	0.514
14	Sway area (cm^2/s)	0.08	0.01	0.12	0.01	0.162
15	Major (cm)	0.94	0.06	1.07	0.09	0.611
16	Minor (cm)	0.42	0.04	0.47	0.03	0.233
17	Eccentricity	0.86	0.02	0.87	0.01	0.926
18	MFREQ AP (Hz)	0.34	0.03	0.45	0.03	0.009
19	MFREQ ML (Hz)	0.51	0.03	0.47	0.03	0.401
20	F-dim	1.52	0.02	1.58	0.01	0.015
21	SD of COMacc AP (cm/s^2)	0.86	0.04	1.28	0.06	< 0.001
22	SD of COMscc ML (cm/s^2)	0.74	0.05	0.76	0.03	0.367
23	Slope-LF AP	-1.75	0.08	-1.36	0.07	0.002
24	Slope-HF AP	-3.36	0.21	-3.77	0.11	0.039
25	Slope-LF ML	-1.21	0.08	-1.32	0.07	0.305
26	Slope-HF ML	-2.98	0.19	-3.19	0.12	0.487
27	Intersect-PSD AP (Hz)	0.92	0.13	0.95	0.04	0.611
28	Intersect-PSD ML (Hz)	0.73	0.09	0.80	0.04	0.425
29	PF50 AP (mm^2/Hz)	0.16	0.02	0.23	0.02	0.038
30	PF50 ML (mm^2/Hz)	0.19	0.03	0.24	0.02	0.137

No.	Name	Normal				
		Mean (Young)	SE (Young)	Mean (Elderly)	SE (Elderly)	p-value
31	PF95 AP (mm^2/Hz)	0.34	0.04	0.53	0.05	0.015
32	PF95 ML (mm^2/Hz)	0.58	0.06	0.52	0.05	0.460
33	CF AP (Hz)	0.61	0.02	0.67	0.02	0.162
34	CF ML (Hz)	0.90	0.05	0.74	0.02	0.005
35	FD AP	0.65	0.01	0.62	0.01	0.104
36	FD ML	0.63	0.01	0.63	0.01	0.641
37	D-short AP (mm^2/s)	0.05	0.00	0.10	0.01	< 0.001
38	D-long AP (mm^2/s)	0.01	0.00	0.02	0.01	0.717
39	Critical-Δtime AP (s)	1.26	0.09	1.10	0.08	0.070
40	Critical-mm AP (mm^2)	0.12	0.02	0.21	0.03	0.013
41	D-short ML (mm^2/s)	0.02	0.00	0.02	0.00	0.181
42	D-long ML (mm^2/s)	0.00	0.00	0.00	0.00	0.104
43	Critical-Δtime ML (s)	0.63	0.16	0.96	0.07	0.091
44	Critical-mm ML (mm^2)	0.02	0.01	0.04	0.01	0.055
45	Hr-short AP (mm^2/s)	0.75	0.01	0.73	0.01	0.487
46	Hr-long AP (mm^2/s)	0.21	0.02	0.16	0.03	0.133
47	Intersect-Δtime AP (s)	-0.12	0.02	-0.17	0.03	0.241
48	Intersect-mm AP (mm^2)	-1.13	0.06	-0.89	0.05	0.008
49	Hr-short ML (mm^2/s)	0.66	0.01	0.71	0.01	0.007
50	Hr-long ML (mm^2/s)	0.29	0.03	0.17	0.02	0.001
51	Intersect-Δtime ML (s)	-0.47	0.09	-0.23	0.04	0.004
52	Intersect-mm ML (mm^2)	-2.05	0.13	-1.66	0.08	0.024
53	MT3 (sec)	0.73	0.01	0.71	0.01	0.158
54	MP3	8.72	0.93	7.39	0.65	0.295
55	MD3 (cm)	0.53	0.02	0.70	0.03	< 0.001
56	Mean-cross AP (times)	30.33	2.66	38.10	2.87	0.132
57	Mean-cross ML (times)	41.22	3.47	40.90	3.26	0.811
58	Zero-cross AP (times)	52.28	1.65	61.48	1.76	0.001
59	Zero-cross ML (times)	67.17	2.18	62.10	1.52	0.065
60	Alpha AP	0.65	0.04	0.65	0.04	0.909
61	Beta AP	1.26	0.04	1.30	0.04	0.909
62	Alpha ML	0.57	0.04	0.59	0.03	0.597
63	Beta ML	1.36	0.04	1.34	0.03	0.597

No.	Name	Normal				
		Mean (Young)	SE (Young)	Mean (Elderly)	SE (Elderly)	p-value
64	LNG-area (1/cm)	35.62	3.45	39.45	3.25	0.437
65	Total power AP (cm^2/Hz)	10.25	1.08	18.92	1.97	0.001
66	Total power ML (cm^2/Hz)	2.64	0.49	3.86	0.59	0.104
67	Mean-MT (sec)	0.74	0.01	0.72	0.01	0.188
68	Slope-MP	4.43	0.42	4.03	0.32	0.487
69	Mean-MD (cm)	0.54	0.02	0.70	0.03	< 0.001

No.	Name	Narrow				
		Mean (Young)	SE (Young)	Mean (Elderly)	SE (Elderly)	p-value
1	Range AP (cm)	2.34	0.15	2.73	0.17	0.23
2	Range ML (cm)	2.21	0.17	2.42	0.15	0.60
3	SD of AP sway (cm)	0.46	0.03	0.49	0.03	0.55
4	SD of ML sway (cm)	0.41	0.03	0.43	0.03	0.73
5	Area (cm^2)	2.77	0.29	3.36	0.39	0.64
6	Velocity maximum (cm/s)	5.12	0.46	7.11	0.58	0.005
7	Total distance (cm)	56.54	4.70	79.72	5.67	0.001
8	Area circle (cm^2)	3.53	0.39	4.10	0.49	0.84
9	Velocity AP (cm/s)	0.68	0.06	1.00	0.08	0.001
10	Velocity ML (cm/s)	0.75	0.07	1.02	0.08	0.01
11	Angle (degree)	30.68	6.37	30.08	3.93	0.75
12	SD of Velocity AP (cm/s)	0.89	0.07	1.29	0.10	0.002
13	SD of Velocity ML (cm/s)	1.01	0.09	1.35	0.10	0.01
14	Sway area (cm^2/s)	0.21	0.03	0.32	0.04	0.04
15	Major (cm)	1.29	0.08	1.30	0.08	0.86
16	Minor (cm)	0.80	0.05	0.93	0.05	0.16
17	Eccentricity	0.75	0.03	0.66	0.02	0.01
18	MFREQ AP (Hz)	0.34	0.02	0.46	0.03	0.003
19	MFREQ ML (Hz)	0.43	0.04	0.54	0.03	0.01
20	F-dim	1.55	0.02	1.62	0.01	0.003
21	SD of COMacc AP (cm/s^2)	1.05	0.06	1.49	0.09	< 0.001
22	SD of COMscc ML (cm/s^2)	1.08	0.09	1.51	0.08	0.001
23	Slope-LF AP	-1.53	0.07	-1.36	0.07	0.10

No.	Name	Narrow				
		Mean (Young)	SE (Young)	Mean (Elderly)	SE (Elderly)	p-value
24	Slope-HF AP	-3.58	0.16	-3.50	0.13	0.63
25	Slope-LF ML	-1.21	0.09	-1.11	0.07	0.32
26	Slope-HF ML	-3.97	0.18	-4.10	0.14	0.40
27	Intersect-PSD AP (Hz)	0.87	0.05	0.88	0.04	0.81
28	Intersect-PSD ML (Hz)	0.89	0.07	1.35	0.32	0.16
29	PF50 AP (mm^2/Hz)	0.15	0.02	0.22	0.02	0.01
30	PF50 ML (mm^2/Hz)	0.25	0.03	0.25	0.02	0.76
31	PF95 AP (mm^2/Hz)	0.37	0.05	0.52	0.05	0.05
32	PF95 ML (mm^2/Hz)	0.60	0.06	0.67	0.05	0.43
33	CF AP (Hz)	0.66	0.03	0.68	0.02	0.93
34	CF ML (Hz)	0.69	0.02	0.74	0.02	0.28
35	FD AP	0.63	0.01	0.63	0.01	0.94
36	FD ML	0.60	0.01	0.61	0.01	0.57
37	D-short AP (mm^2/s)	0.08	0.01	0.15	0.02	0.002
38	D-long AP (mm^2/s)	0.02	0.00	0.01	0.00	0.26
39	Critical-Δtime AP (s)	1.09	0.15	1.13	0.08	0.83
40	Critical-mm AP (mm^2)	0.17	0.04	0.30	0.04	0.004
41	D-short ML (mm^2/s)	0.10	0.02	0.15	0.02	0.01
42	D-long ML (mm^2/s)	0.01	0.00	0.01	0.00	0.89
43	Critical-Δtime ML (s)	0.90	0.09	0.96	0.05	0.63
44	Critical-mm ML (mm^2)	0.19	0.05	0.29	0.04	0.02
45	Hr-short AP (mm^2/s)	0.74	0.01	0.73	0.01	0.28
46	Hr-long AP (mm^2/s)	0.24	0.03	0.13	0.02	0.01
47	Intersect-Δtime AP (s)	-0.20	0.04	-0.14	0.02	0.29
48	Intersect-mm AP (mm^2)	-1.06	0.08	-0.69	0.05	0.001
49	Hr-short ML (mm^2/s)	0.73	0.01	0.73	0.01	0.78
50	Hr-long ML (mm^2/s)	0.16	0.04	0.11	0.02	0.50
51	Intersect-Δtime ML (s)	-0.23	0.04	-0.21	0.03	0.96
52	Intersect-mm ML (mm^2)	-0.99	0.10	-0.75	0.05	0.04
53	MT3 (sec)	0.70	0.01	0.66	0.01	0.01
54	MP3	3.82	0.48	3.36	0.26	0.63
55	MD3 (cm)	0.79	0.06	1.02	0.06	0.001
56	Mean-cross AP (times)	28.28	2.64	39.58	2.51	0.01

No.	Name	Narrow				
		Mean (Young)	SE (Young)	Mean (Elderly)	SE (Elderly)	p-value
57	Zero-cross AP (times)	37.67	3.67	47.55	2.69	0.02
58	Zero-cross ML (times)	61.78	1.66	61.13	1.67	0.55
59	Alpha AP	60.56	1.51	66.71	1.75	0.03
60	Beta AP	0.63	0.04	0.62	0.03	0.93
61	Alpha ML	1.29	0.04	1.31	0.03	0.93
62	Beta ML	0.56	0.03	0.51	0.02	0.11
63	Beta ML	1.36	0.04	1.43	0.03	0.11
64	LNG-area (1/cm)	19.08	1.95	22.81	1.46	0.07
65	Total power AP (cm^2/Hz)	15.51	2.42	27.60	3.04	0.003
66	Total power ML (cm^2/Hz)	18.90	4.41	27.49	4.13	0.02
67	Mean-MT (sec)	0.71	0.01	0.67	0.01	0.03
68	Slope-MP	2.34	0.26	2.17	0.17	0.76
69	Mean-MD (cm)	0.80	0.06	1.04	0.06	0.001

No.	Name	Foam				
		Mean (Young)	SE (Young)	Mean (Elderly)	SE (Elderly)	p-value
1	Range AP (cm)	2.98	0.16	4.12	0.23	0.001
2	Range ML (cm)	1.63	0.12	1.95	0.13	0.07
3	SD of AP sway (cm)	0.57	0.03	0.75	0.04	0.003
4	SD of ML sway (cm)	0.30	0.02	0.35	0.02	0.28
5	Area (cm^2)	2.63	0.28	4.17	0.51	0.03
6	Velocity maximum (cm/s)	6.71	0.61	9.50	0.48	< 0.001
7	Total distance (cm)	53.32	1.99	89.24	3.78	< 0.001
8	Area circle (cm^2)	3.93	0.42	6.86	0.90	0.003
9	Velocity AP (cm/s)	0.81	0.03	1.53	0.07	< 0.001
10	Velocity ML (cm/s)	0.52	0.03	0.65	0.04	0.04
11	Angle (degree)	9.15	2.06	6.85	1.67	0.44
12	SD of Velocity AP (cm/s)	1.09	0.04	2.02	0.08	< 0.001
13	SD of Velocity ML (cm/s)	0.70	0.04	0.85	0.05	0.06
14	Sway area (cm^2/s)	0.18	0.01	0.33	0.03	< 0.001
15	Major (cm)	1.43	0.09	1.85	0.10	0.01
16	Minor (cm)	0.69	0.04	0.83	0.05	0.16

No.	Name	Foam				
		Mean (Young)	SE (Young)	Mean (Elderly)	SE (Elderly)	p-value
17	Eccentricity	0.85	0.02	0.87	0.02	0.46
18	MFREQ AP (Hz)	0.33	0.02	0.48	0.02	< 0.001
19	MFREQ ML (Hz)	0.41	0.03	0.44	0.02	0.19
20	F-dim	1.51	0.01	1.57	0.01	0.01
21	SD of COMacc AP (cm/s^2)	1.22	0.07	2.32	0.09	< 0.001
22	SD of COMscc ML (cm/s^2)	0.87	0.06	1.10	0.05	0.004
23	Slope-LF AP	-1.75	0.05	-1.39	0.08	0.003
24	Slope-HF AP	-3.82	0.15	-3.47	0.15	0.21
25	Slope-LF ML	-1.47	0.11	-1.53	0.09	0.83
26	Slope-HF ML	-2.96	0.21	-3.05	0.10	0.66
27	Intersect-PSD AP (Hz)	0.91	0.07	1.00	0.12	0.98
28	Intersect-PSD ML (Hz)	0.73	0.04	0.75	0.07	0.88
29	PF50 AP (mm^2/Hz)	0.18	0.01	0.24	0.01	0.002
30	PF50 ML (mm^2/Hz)	0.24	0.03	0.25	0.02	0.91
31	PF95 AP (mm^2/Hz)	0.35	0.03	0.53	0.04	0.001
32	PF95 ML (mm^2/Hz)	0.52	0.05	0.48	0.04	0.51
33	CF AP (Hz)	0.54	0.02	0.62	0.02	0.02
34	CF ML (Hz)	0.70	0.04	0.59	0.02	0.01
35	FD AP	0.61	0.01	0.64	0.01	0.15
36	FD ML	0.62	0.01	0.62	0.01	0.83
37	D-short AP (mm^2/s)	0.16	0.02	0.41	0.03	< 0.001
38	D-long AP (mm^2/s)	0.02	0.00	0.01	0.01	0.16
39	Critical-Δtime AP (s)	1.27	0.08	1.16	0.05	0.33
40	Critical-mm AP (mm^2)	0.37	0.04	0.92	0.08	< 0.001
41	D-short ML (mm^2/s)	0.04	0.01	0.09	0.01	0.004
42	D-long ML (mm^2/s)	0.01	0.00	0.00	0.00	0.08
43	Critical-Δtime ML (s)	0.91	0.09	1.17	0.07	0.05
44	Critical-mm ML (mm^2)	0.08	0.01	0.20	0.03	0.002
45	Hr-short AP (mm^2/s)	0.78	0.01	0.74	0.01	0.03
46	Hr-long AP (mm^2/s)	0.15	0.03	0.05	0.02	0.01
47	Intersect-Δtime AP (s)	-0.07	0.03	-0.06	0.02	0.94
48	Intersect-mm AP (mm^2)	-0.58	0.07	-0.12	0.04	< 0.001
49	Hr-short ML (mm^2/s)	0.74	0.01	0.77	0.01	0.08

No.	Name	Foam				
		Mean (Young)	SE (Young)	Mean (Elderly)	SE (Elderly)	p-value
50	Hr-long ML (mm^2/s)	0.22	0.03	0.10	0.01	0.001
51	Intersect-Δtime ML (s)	-0.25	0.05	-0.08	0.02	0.002
52	Intersect-mm ML (mm^2)	-1.36	0.10	-0.88	0.06	< 0.001
53	MT3 (sec)	0.69	0.02	0.64	0.01	0.01
54	MP3	3.73	0.35	2.68	0.24	0.01
55	MD3 (cm)	0.73	0.02	1.13	0.04	< 0.001
56	Mean-cross AP (times)	28.56	1.75	42.84	2.55	< 0.001
57	Mean-cross ML (times)	32.06	3.11	38.77	2.52	0.15
58	Zero-cross AP (times)	48.83	1.68	55.26	1.63	0.01
59	Zero-cross ML (times)	57.17	1.67	52.39	1.54	0.04
60	Beta AP	0.80	0.06	0.70	0.04	0.15
61	Alpha ML	1.15	0.04	1.23	0.03	0.15
62	Beta ML	0.62	0.04	0.69	0.03	0.13
63	Beta ML	1.30	0.04	1.23	0.03	0.13
64	LNG-area (1/cm)	19.92	2.11	21.97	1.63	0.32
65	Total power AP (cm^2/Hz)	33.36	3.49	87.87	8.02	< 0.001
66	Total power ML (cm^2/Hz)	7.41	0.85	17.61	2.45	0.001
67	Mean-MT (sec)	0.72	0.01	0.66	0.01	0.001
68	Slope-MP	2.32	0.23	1.68	0.15	0.03
69	Mean-MD (cm)	0.76	0.03	1.16	0.04	< 0.001

No.	Name	Foam				
		Mean (Young)	SE (Young)	Mean (Elderly)	Mean (Young)	p-value
1	Range AP (cm)	2.33	0.18	2.72	0.21	0.28
2	Range ML (cm)	1.08	0.06	1.57	0.37	0.66
3	SD of AP sway (cm)	0.46	0.03	0.47	0.03	0.93
4	SD of ML sway (cm)	0.21	0.02	0.24	0.03	0.98
5	Area (cm^2)	1.27	0.13	1.92	0.34	0.47
6	Velocity maximum (cm/s)	5.41	0.43	7.85	1.48	0.07
7	Total distance (cm)	39.72	1.76	66.12	4.13	< 0.001
8	Area circle (cm^2)	2.53	0.31	3.26	0.59	0.89
9	Velocity AP (cm/s)	0.60	0.03	1.11	0.08	< 0.001

No.	Name	EC				
		Mean (Young)	SE (Young)	Mean (Elderly)	SE (Elderly)	p-value
10	Velocity ML (cm/s)	0.40	0.02	0.50	0.03	0.02
11	Angle (degree)	14.26	2.27	11.22	2.64	0.05
12	SD of Velocity AP (cm/s)	0.78	0.04	1.45	0.10	< 0.001
13	SD of Velocity ML (cm/s)	0.53	0.02	0.71	0.07	0.04
14	Sway area (cm^2/s)	0.10	0.01	0.18	0.02	0.04
15	Major (cm)	1.17	0.08	1.19	0.09	0.89
16	Minor (cm)	0.42	0.02	0.54	0.05	0.19
17	Eccentricity	0.92	0.01	0.87	0.02	0.02
18	MFREQ AP (Hz)	0.30	0.02	0.57	0.03	< 0.001
19	MFREQ ML (Hz)	0.44	0.03	0.54	0.03	0.09
20	F-dim	1.50	0.02	1.60	0.02	< 0.001
21	SD of COMacc AP (cm/s^2)	0.93	0.05	1.64	0.09	< 0.001
22	SD of COMscc ML (cm/s^2)	0.74	0.03	1.00	0.11	0.01
23	Slope-LF AP	-1.81	0.09	-1.17	0.07	< 0.001
24	Slope-HF AP	-3.52	0.19	-3.78	0.15	0.18
25	Slope-LF ML	-1.30	0.08	-1.04	0.09	0.07
26	Slope-HF ML	-3.03	0.23	-3.28	0.13	0.23
27	Intersect-PSD AP (Hz)	6.43	5.50	1.02	0.07	0.93
28	Intersect-PSD ML (Hz)	160.43	159.75	0.81	0.05	0.32
29	PF50 AP (mm^2/Hz)	0.18	0.02	0.26	0.02	0.01
30	PF50 ML (mm^2/Hz)	0.25	0.03	0.30	0.03	0.40
31	PF95 AP (mm^2/Hz)	0.36	0.04	0.67	0.05	< 0.001
32	PF95 ML (mm^2/Hz)	0.53	0.06	0.65	0.05	0.18
33	CF AP (Hz)	0.58	0.02	0.75	0.03	< 0.001
34	CF ML (Hz)	0.80	0.03	0.80	0.02	0.93
35	FD AP	0.65	0.01	0.62	0.01	0.17
36	FD ML	0.64	0.01	0.61	0.01	0.14
37	D-short AP (mm^2/s)	0.07	0.01	0.18	0.02	< 0.001
38	D-long AP (mm^2/s)	0.01	0.00	0.02	0.01	0.21
39	Critical-Δtime AP (s)	1.30	0.11	0.92	0.05	0.004
40	Critical-mm AP (mm^2)	0.17	0.03	0.32	0.04	0.01
41	D-short ML (mm^2/s)	0.02	0.00	0.05	0.02	0.03
42	D-long ML (mm^2/s)	0.00	0.00	0.04	0.04	0.29

No.	Name	EC				
		Mean (Young)	SE (Young)	Mean (Elderly)	SE (Elderly)	p-value
43	Critical-Δtime ML (s)	0.71	0.14	0.78	0.06	0.91
44	Critical-mm ML (mm^2)	0.02	0.01	0.06	0.01	0.02
45	Hr-short AP (mm^2/s)	0.76	0.01	0.73	0.01	0.12
46	Hr-long AP (mm^2/s)	0.20	0.02	0.11	0.02	0.003
47	Intersect-Δtime AP (s)	-0.10	0.03	-0.21	0.02	0.01
48	Intersect-mm AP (mm^2)	-0.99	0.07	-0.71	0.06	0.004
49	Hr-short ML (mm^2/s)	0.67	0.01	0.69	0.01	0.19
50	Hr-long ML (mm^2/s)	0.27	0.03	0.18	0.02	0.01
51	Intersect-Δtime ML (s)	-0.40	0.06	-0.30	0.04	0.15
52	Intersect-mm ML (mm^2)	-1.96	0.11	-1.56	0.11	0.02
53	MT3 (sec)	0.74	0.02	0.68	0.01	0.01
54	MP3	7.14	0.58	7.10	0.78	0.38
55	MD3 (cm)	0.58	0.03	0.89	0.05	< 0.001
56	Mean-cross AP (times)	23.33	1.94	51.26	3.36	< 0.001
57	Mean-cross ML (times)	36.28	3.68	47.45	2.79	0.01
58	Zero-cross AP (times)	56.11	2.02	67.48	1.75	< 0.001
59	Zero-cross ML (times)	62.72	2.28	66.45	1.24	0.24
60	Beta AP	0.75	0.05	0.61	0.02	0.04
61	Alpha ML	1.19	0.04	1.30	0.03	0.04
62	Beta ML	0.51	0.03	0.54	0.02	0.46
63	Beta ML	1.44	0.05	1.38	0.03	0.46
64	LNG-area (1/cm)	29.25	2.40	44.52	4.42	0.05
65	Total power AP (cm^2/Hz)	14.76	2.12	30.38	3.91	0.003
66	Total power ML (cm^2/Hz)	2.80	0.37	13.40	8.41	0.07
67	Mean-MT (sec)	0.75	0.01	0.69	0.01	0.001
68	Slope-MP	3.78	0.28	3.94	0.36	0.80
69	Mean-MD (cm)	0.59	0.03	0.89	0.05	< 0.001

AP, Anteroposterior direction; ML, Mediolateral direction; COP, Center of Pressure; COM, Center of Mass
